# Dynamic Regulation of TCR–Microclusters and the Microsynapse for T Cell Activation

**DOI:** 10.3389/fimmu.2016.00255

**Published:** 2016-06-28

**Authors:** Akiko Hashimoto-Tane, Takashi Saito

**Affiliations:** ^1^Laboratory for Cell Signaling, RIKEN Center for Integrative Medical Sciences, Yokohama, Japan

**Keywords:** immunological synapse, microcluster, microsynapse, dynein, cSMAC, cytoskeleton, F-actin, LFA1

## Abstract

The interaction between a T cell and an antigen-presenting cell is the initiating event in T cell-mediated adaptive immunity. The Immunological Synapse (IS) is formed at the interface between these two cell types, and is the site where antigen (Ag)-specific recognition and activation are induced through the T cell receptor (TCR). This occurs at the center of the IS, and cell adhesion is supported through integrins in the area surrounding the TCR. Recently, this model has been revised based on data indicating that the initial Ag-specific activation signal is triggered prior to IS formation at TCR–microclusters (MCs), sites where TCR, kinases and adaptors of TCR proximal downstream signaling molecules accumulate as an activation signaling cluster. TCR–MCs then move into the center of the cell–cell interface to generate the cSMAC. This translocation of TCR–MCs is mediated initially by the actin cytoskeleton and then by dynein-induced movement along microtubules. The translocation of TCR–MCs and cSMAC formation is induced upon strong TCR stimulation through the assembly of a TCR–dynein super complex with microtubules. The Ag-specific activation signal is induced at TCR–MCs, but the adhesion signal is now shown to be induced by generating a “microsynapse,” which is composed of a core of TCR–MCs and the surrounding adhesion ring of integrin and focal adhesion molecules. Since the microsynapse is critical for activation, particularly under weak TCR stimulation, this structure supports a weak TCR signal through a cell–cell adhesion signal. The microsynapse has a structure similar to the IS but on a micro-scale and regulates Ag-specific activation as well as cell–cell adhesion. We describe here the dynamic regulation of TCR–MCs, responsible for inducing Ag-specific activation signals, and the microsynapse, responsible for adhesion signals critical for cell–cell interactions, and their interrelationship.

## TCR–Microclusters and the Immune Synapse

### T Cell Activation Signaling through TCR–Microclusters

Acquired immunity is exemplified by antigen (Ag)-specific responses, which are initiated by specific recognition of Ag by T or B cells. In the case of T cells, an Ag-specific cell encounters and interacts with Ag-bearing dendritic cell (DC) in the draining lymph node and uses its T cell receptor (TCR) to recognize the Ag peptide–MHC complex on the DC. This interaction induces an Immunological Synapse (IS) at the interface between the T cell and DC. The initial finding of this structure by Kupfer’s group was mainly based on microscopic visualization of the *z*-axis of the interface using advanced microscopy and deconvolution. They observed that the interface had a specifically segregated bulls-eye structure with a centralized TCR (with PKCθ) surrounded by the integrin LFA-1 (with Talin) (1). This segregation was achieved by accumulation of TCRs and adhesion molecules together with certain signaling molecules. Thus, the structure appeared to be related to T cell activation, and they termed the structure, the Supra Molecular Activation Cluster (SMAC), the central area for TCR accumulation as the cSMAC, and the peripheral LFA-1 accumulated area as the pSMAC. The initial analysis already noted the size difference of molecules in the c- and p-SMAC, i.e., that smaller molecules with one or two Ig domains in their extracellular region tended to accumulate in the cSMAC, while larger molecules such as integrins or CD45 accumulated in the pSMAC or distal dSMAC surrounding the cSMAC (2). These observations led to the segregation kinetics model of T cell activation (3), which proposes that the segregation in the periphery of large sized phosphatases such as CD45 from the central region of TCR engagement allows for activation of Lck kinase, followed by induction of the initial downstream signals for T cell activation (4).

The mature IS, supported by cellular adhesion through LFA-1, was thought to be an ideal structure for inducing an activation signal upon Ag recognition through the TCR. However, it was noticed that IS formation can be variable, depending on the cell types and stimulation conditions; some T cells do not form IS, nor do T cells with B cells rather than DC as antigen-presenting cell (APC) (5, 6). It was also proposed that only strong stimulation induced cSMAC formation (these situations are discussed later in terms of their relationship with the microsynapse). Furthermore, since the generation of the cSMAC, even on a supported planar bilayer, took about 10 min after interaction of the TCR and pMHC, it was noted that this would be too late for triggering the initial TCR signals (7). Analysis of very early activation after the interaction of Ag-specific T cells and a planar bilayer containing specific Ag peptide–MHC revealed that the TCR begins clustering immediately after T cells recognize the peptide–MHC on the planar bilayer, prior to mature IS formation. We stated to call these initial clusters TCR–microclusters (TCR–MC) as a minimal unit of clusters mediating both initial and sustained TCR signaling (8–12). MCs were described by Krummel and Davis as small clusters of CD3ζ accumulating at the center of the interface upon stimulation, and which were synchronized with the calcium response (13, 14). Quantification analysis of the TCR–MCs revealed that each cluster contains approximately one hundred (50–300) TCR molecules. This TCR accumulation immediately upon peptide/MHC stimulation was found to be associated with the simultaneous accumulation of the kinase ZAP70 and adaptor proteins LAT and SLP76 in the same TCR–MC. Upon stimulation, every TCR–MC is stained by Abs against phospho-ZAP70, phospho-SLP-76, and phospho-tyrosine. Thus, a TCR–MC is generated by accumulation of a hundred TCR–CD3 complexes, kinases and adaptors and induces immediate phosphorylation of these molecules. TCR–MCs also contain a substantial quantity of the known proximal signaling intermediates including ZAP70, LAT, SLP-76, PLCγ, and cytoskeleton-related molecules Nck and Vav (15, 16), which further induce triggering of a Ca^2+^ flux and activation of downstream effector molecules. TCR–MCs are generated first at the center of the interface between the T cell and the planar bilayer or APC, and then are increased over the entire interface as the T cells spread. The initial activation signal is therefore induced in the newly generated TCR–MCs on the cell surface. Regarding the relationship of TCR–MC and the IS, TCR–MCs move toward the center of the interface after maximum cellular spreading, and the accumulated TCRs generate the cSMAC of the IS. It was noticed that only the TCR–CD3 complexes move to the center to form the cSMAC, while other signaling molecules such as ZAP70 and SLP-76 move only a short distance toward the center but do not accumulate in the cSMAC. These molecules disappear during their transport toward the center, probably by endocytosis. It has been noticed that TCR–MCs do not generate a cSMAC in some T cells, such as thymocytes and hybridomas, or under certain conditions, including weak Ag stimulation. However, even under conditions without cSMAC formation, T cells generate TCR–MCs to induce activation signals.

Signaling clusters induced upon TCR stimulation had been previously demonstrated when Jurkat cells were stimulated by immobilized anti-CD3 Ab (15). In this situation, TCR-CD3 appeared to be fixed and immobilized on the cover slip, but clusters of LAT, SLP76, and PLCγ, which induce the phosphorylation and activation of downstream signaling molecules, were generated. In this system, distal signaling intermediate molecules become dissociated from the immobilized TCR and move to intracellular compartments; SLP76 moves to a perinuclear structure and Nck and WASP to an actin-rich compartment and the immobilized TCRs do not move to the center or make the cSMAC (15, 16). Although there are some differences between pMHC-induced TCR–MC in normal T cells and Ab-stimulated signaling clusters in Jurkat cells, a general common feature is that, prior to the IS formation, TCR–MCs composed of TCR-CD3, kinases and adaptors are generated at the interface upon Ag recognition, which induces the initial signal for T cell activation. Later, though depending on stimulation conditions, the TCR–MCs move to the center of the interface to generate the cSMAC of the mature IS.

Recent imaging analysis using super-resolution microscopy as well as EM analysis revealed that several molecules of TCR or LAT are pre-clustered prior to Ag stimulation as “nanoclusters,” which are then assembled together upon stimulation to form a MC (17, 18). In this regard, it is noted that the dynamics of signaling molecules within TCR–MCs are not uniformly regulated, and the signaling components within the cluster are variable and dynamic in their behavior.

A transient initial activation signal is not sufficient for full activation of T cells to induce cytokine production and cell proliferation, instead sustained activation for several hours is at a minimum required (19). Not only initial activation but also sustained continuous activation is induced through TCR–MCs at the peripheral region of the interface (9). TCR–MCs are continuously generated at the cellular edge with lamellipodial structures and move inward to the cSMAC. When the generation of the peripheral MCs is interrupted by the addition of anti-pMHC Ab, the formation of peripheral TCR–MCs is immediately halted, but the cSMAC is maintained (10). Moreover, the blockade of newly generated TCR–MCs functionally inhibited activation signals. Therefore, the continuous generation of TCR–MCs is critical for inducing sustained activation signals.

### Compartmentalization of TCR Signaling at the IS

The cSMAC, as the representative structure of the original description of the IS, has several specific functions: (a) increasing the cell adhesion between the T cell and APC. Since the affinity of the individual TCR and pMHC interaction is so low, on the order of 10^−4^M (20), the accumulation of thousands of TCRs increases the avidity for pMHC for cellular adhesion between the T cell and APC, although the adhesion is mainly supported by integrin binding in the pSMAC. (b) directing the targeted secretion of cytotoxic granules and cytokines toward APC (21, 22). (c) serving as the site for endocytosis and/or exocytosis (23, 24) of the TCR complex, which functions to negatively regulate T cell activation. (d) inducing co-stimulation signals as described below.

In the case of the IS formed between cytotoxic T cells and target cells, the cSMAC area is further segregated into two functional domains, a signaling domain through the TCR and a secretory domain, through which cytotoxic granules are secreted onto target cells (23, 25). These functional domains are present even in CD4^+^ T cells.

**Figure 1:**
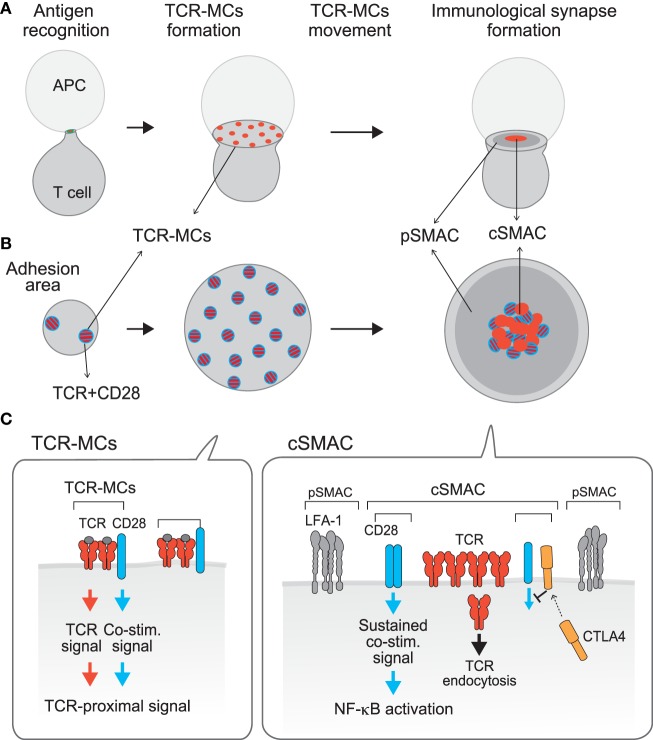
**Compartmentalization of TCR activation signals: TCR–microcluster and cSMAC**. [**(A)**, *x*–*y* axis; **(B)**, *z*-axis; **(C)**, a model for the assembly on the membrane] Upon Ag recognition, T cells form a conjugate with the APC (left) and generate TCR–microclusters (MCs) at the interface with the APC. TCR–MCs contain TCRs (red) and the proximal signaling molecules as well as the CD28 co-stimulation receptor (blue), and induce the initial activation signal (middle). After maximum spreading, TCR–MCs begin to move toward the center of the interface to form the cSMAC (right). It was noted that there is a CD3^hi^ region (red) and a CD3^lo^ region (mixture of red and blue) within the cSMAC; the CD3^hi^ region is rigid and may represent the site for TCR endocytosis, whereas the CD3^lo^ region is dynamically regulated and various costimulation molecules as CD28 and CTLA-4 are co-localized. Thus, we named this CD3^lo^ region the “signaling cSMAC.” In the cSMAC, the TCR complex is subjected to endocytosis/degradation for negative regulation, whereas CD28 recruits PKCθ and CARMA1 to induce a sustained co-stimulation signal leading to downstream events such as of NF-κB activation. The inhibitory co-stimulation receptor CTLA4 is translocated to the same cSMAC area as CD28 and competes with CD28 to eject CD28-PKCθ from the cSMAC, resulting in inhibition of activation. Thus, the TCR activation signal is regulated by spatially distinct signals: The Ag recognition signal as “Signal 1” is mediated by TCR–MCs and a sustained co-stimulation signal as “Signal 2” is mediated by the signaling region of the cSMAC.

Because the TCR/CD3 complex accumulates in the cSMAC, whereas upon Ag recognition, the majority of the downstream kinases and adaptors do not (9, 10), and little phosphorylation of these signaling molecules was observed in the cSMAC, it is thought to be inactive in signaling. Rather, the cSMAC is thought to be responsible for endocytosis and degradation of the TCR, which consequently contributes to negative regulation of T cell activation by decreasing the TCR complex. Indeed, a large invagination of the TCR was observed (26), and endocytic/degradation machinery such as TSG101 (27) and a lipid for multivesicular body formation for degradation, lysobisphosphatidic acid (LBPA), is assembled in the cSMAC (10), indicating that the TCR complex is endocytosed at the cSMAC and targeted for degradation. In contrast to its function in TCR endocytosis, it was recently reported that vesicles containing the TCR are secreted from the cSMAC (24). Thus, the contribution of endocytosis vs. exocytosis of TCR-containing vesicles in the cSMAC has to be better understood. When the cSMAC area was precisely analyzed by microscopy, two distinct areas were found – CD3^hi^ and CD3^lo^ (Figure 1) Bleaching experiments revealed that the CD3^hi^ area is rigid whereas the CD3^lo^ area is very mobile and dynamically regulated. Using planar membranes containing MHC class II with covalently linked peptide, the CD3^lo^ area but not the CD3^hi^ region was found to be associated with pMHC, suggesting that the CD3^lo^ region is actively participating in binding to pMHC, but the CD3^hi^ region may contain TCR complexes that are either in the process of dissociation from pMHC or have already done so and are on the path to endocytosis and/or exocytosis (28, 29).

In contrast to these data showing that the cSMAC is in general a negative regulatory site through TCR endocytosis/degradation, imaging analysis of co-stimulation signals indicated that a sustained co-stimulation signal is induced through a part of the cSMAC (30, 31). We demonstrated that upon Ag stimulation, the positive co-stimulatory receptor CD28 is first co-localized in the peripheral TCR–MC (recall that a co-stimulation signal is induced through the TCR–MC in the initial phase of activation) and then moves to and later accumulates in the cSMAC region, particularly in the CD3^lo^ area (we call this area the “signaling cSMAC”) (Figure 1). A search for the molecules whose behavior is similar to CD28 identified PKCθ and CARMA1, which also accumulated in a similar region of the cSMAC. A CTLA4–Ig fusion protein is used to inhibit CD28 co-stimulation since it binds more strongly than CD28 to the common ligand CD80/86. Addition of CTLA4–Ig blocks the association between CD28 and its ligands and results in no accumulation of CD28 in the signaling cSMAC. At the same time, PKCθ was also no longer found in the cSMAC, indicating that CD28 recruits PKCθ to the signaling cSMAC, probably to mediate co-stimulation. Using a biochemical approach, CD28 was found to be physically associated with PKCθ through association with Lck. The V3 region of PKCθ binds to the SH3 region of Lck and the SH2 region of Lck binds to the proline-rich region of CD28 (32). The accumulated CD28 recruits PKCθ and then CARMA1 into the CD3^lo^ signaling cSMAC region, where sustained co-stimulation signals are induced, including NF-κB activation.

The analysis of TCR–MC and the cSMAC has revealed spatially distinct signaling compartments within a single T cell. These might be structural correlates corresponding to the old idea that both signal 1 and signal 2 are required for full T cell activation, i.e., TCR-induced Ag-recognition signal as signal 1 is mediated through the TCR–MC whereas the CD28-induced sustained co-stimulation signal as signal 2 is mediated through the signaling cSMAC (Figure 1). Recently, an actin-uncapping protein Rltpr was shown to be essential for CD28-mediated co-stimulation, a finding that connects CD28 and PKCθ/CARMA1 (33). Rltpr is also localized in the same signaling cSMAC upon TCR stimulation, where it may mediate the co-stimulation signaling function.

Negative regulation of T cell activation by the inhibitory co-stimulation receptor CTLA-4 is also induced at the same cSMAC region. Because CD28 and CTLA4 share the same ligands CD80/CD86 and CTLA4 has a much higher (20-fold) affinity for these ligands, even low expression of CTLA4 on the T cell surface can compete with CD28 for ligand binding, which is the major mechanism for CTLA4-mediated inhibition (34, 35). CTLA-4 mostly accumulates in the intracellular secretory lysosomes and, upon TCR stimulation (36), it moves toward the plasma membrane at the cSMAC, particularly to the CD3^lo^ signaling cSMAC, the same region where CD28 accumulates. Accumulated CTLA4 at the signaling cSMAC locally competes with CD28 for ligand binding. Therefore, CTLA-4-mediated inhibition is induced by ejecting the CD28-PKCθ–CARMA1 signaling machinery from the signaling cSMAC (11).

Thus, the current view of the cSMAC has evolved. It is not merely a site for negative regulation through TCR endocytosis/degradation, but instead a particular region within the cSMAC is the site for inducing activation signals and is also a regulatory site by virtue of its inhibitory co-stimulation.

## Cytoskeletal Regulation of TCR–Microcluster Movement

When T cells are stimulated with different concentrations of peptide/MHC on a planar bilayer, the cSMAC is formed with relatively high concentrations of antigen (>1 μM) but cannot be formed with low concentrations (<10 nM). Stimulation with low doses of Ag induces TCR–MCs, but they do not move toward the center of the interface and do not form the cSMAC. Considering that the cSMAC negatively regulates T cell activation through TCR endocytosis/degradation, weak stimulation to trigger weak signals may not require such an inhibitory mechanism. In contrast, upon strong stimulation with high doses of Ag, TCR–MCs move to and accumulate in the center, generating a cSMAC. Therefore, the movement of TCR–MCs is regulated by activation signal strength. At the steady state before stimulation, the TCR forms small clusters on the cell surface consisting of a few to ten molecules as “nano-clusters” (17, 37), as described above. Some signaling molecules such as LAT have been shown to be in nano-clusters distinct from the TCR. However, upon TCR stimulation, these distinct nano-clusters begin to form larger clusters by coalescing with signaling molecules such as LAT (17). These coalesced clusters are likely to be equivalent to TCR–MCs as a signaling unit. The size of TCR–MCs to be translocated centripetally into the cSMAC should be minimum (38).

**Figure 2:**
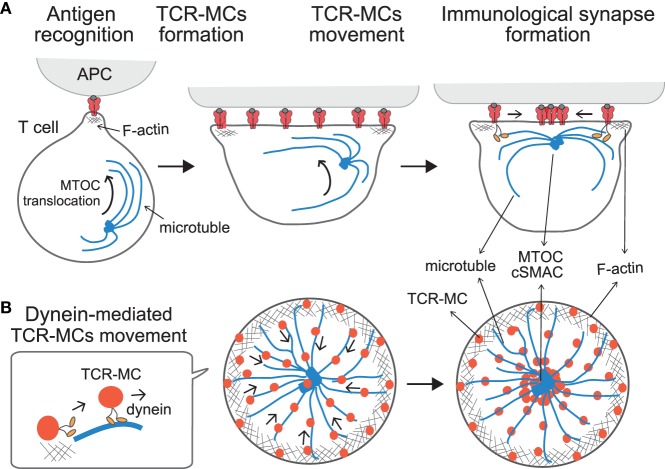
**Translocation of TCR–microclusters through cytoskeletal regulation**. **(A,B)** depict the *x*–*y* axis and the *z* axis, respectively. Upon Ag recognition, T cells generate TCR–MCs (red) all over the interface. During this time, the MTOC (blue dot) is quickly translocated to the vicinity to the plasma membrane and finally to the TCR engagement site. Initially, TCR–MCs generated in the peripheral area move toward the center, coincident with actin retrograde flow (mesh structure). Thereafter, TCR–MCs are translocated to the cSMAC along the microtubules (blue line), which are translocated together with the MTOC into close proximity to the membrane in a dynein-mediated manner. The TCR/CD3 complex associates with the dynein–dynactin complex upon TCR stimulation, and then further assembles with microtubules. The dynein-mediated translocation of TCR–MCs regulates T cell activation because blockade of microtubules or dynein function prevents cSMAC formation and enhances T cell activation.

The translocation and function of TCR–MCs is dependent on the actin cytoskeleton. Upon TCR stimulation, the actin cytoskeleton dynamically changes the cell morphology to promote centripetal flow at the periphery (39, 40). Inhibition of actin polymerization at the initial stage of T cell activation resulted in blockade of T cell adhesion, generation of TCR–MCs and activation. Addition of the actin inhibitor during the initial formation of TCR–MCs inhibits the generation of additional TCR–MCs, and consequently inhibits activation signals (8, 10). F-actin is initially generated upon TCR activation as a distal ring at the peripheral edge of the cell along with cell spreading, and then forms a large peripheral ring. In addition to this distal lamellipodial ring, small foci of F-actin have been found in co-localization with TCR–MCs (41). The peripheral actin exhibits retrograde flow toward the center of the interface. The new TCR–MCs, which are generated at the lamellipodial edge in a random manner upon interaction with peptide–MHC, then start to move toward the center, coincident with the actin retrograde flow (42). Since the interaction of TCR–MCs and actin appears to be weak and the actin centripetal flow is faster than the movement of TCR–MCs, TCR–MCs may be propelled by transient linkage to the actin retrograde flow (42, 43). However, the actin retrograde flow can only reach to about the middle of the path to the center, and the central/peripheral areas are free of actin (44). This raises the question of how are TCR–MCs translocated further to the cSMAC? We found that TCR–MCs translocate further into the central region along microtubules by assembly with the microtubule-associated motor protein dynein (45) (Figure 2). Dynein generally transports various cellular cargos by walking along cytoskeletal microtubules toward the minus-end of the microtubule. Indeed, we could co-immunoprecipitate the dynein–dynactin complex with the TCR complex upon TCR stimulation. When T cells were treated to (a) down-modulate dynein expression by siRNA-mediated knockdown, (b) inhibit dynein kinase activity, or (c) inhibit microtubule formation, TCR–MCs failed to move to the center and did not form the cSMAC. Consequently, these treatments resulted in augmented activation signals, resulting in enhanced phosphorylation of downstream signal molecules, such as SLP76, Vav and Erk, and elevated cytokine production. The finding that inhibition of cSMAC formation resulted in augmented activation indicates that the cSMAC functions as negative regulator, as previously shown similarly in CD2AP-deficient mice (46).

T cell receptor stimulation induces two events in relation to dynein-mediated translocation of TCR–MCs; one is the assembly of the TCR complex with the dynein/dynactin complex, and the other is the translocation of the MTOC (microtubule organization center, or centrosome) to the vicinity to the engagement site on the membrane at the interface. Kinetic studies revealed that MTOC translocation takes place first, followed by the translocation of TCR–MCs (45). Thus, TCR–MCs move along the microtubules, which are localized close to the plasma membrane after the MTOC moves to the site of TCR engagement. The TCR complex is assembled with the dynein complex and associates with microtubules after the MTOC and microtubules become localized to the membrane. This interplay leads the movement of the TCR–MCs toward the center along microtubules in a dynein-mediated fashion, thus generating the cSMAC. Therefore, the translocation of TCR–MCs is regulated cooperatively both through F-actin retrograde flow initially, and then later by dynein-mediated movement along microtubules, ultimately leading to formation of the cSMAC (Figure 2).

The translocation of the MTOC to the interface of the TCR engagement site is regulated by TCR signals upon pMHC stimulation (22, 47, 48). TCR engagement upon triggering with weak stimulation induces neither MTOC translocation nor the translocation of the TCR–MCs to generate the cSMAC. Such a weak stimulatory signal, which is induced at the TCR–MCs, may not need negative regulation at the cSMAC.

## Microsynapses Support Adhesion and Signaling

Ag recognition and subsequent activation of T cells requires strong contact and adhesion with APC for a certain extended time period to induce full activation. Because the affinity of the TCR–pMHC interaction is very low, Ag recognition by the TCR is supported by strong cellular adhesion through specific adhesion molecules, particularly the integrin LFA-1 binding to its ligand ICAM-1/ICAM-2. The TCR-induced activation signal and the LFA1-mediated adhesion signal are mutually regulated. The TCR signal induces a specific LFA-1 conformational change that results in high affinity binding to the ligand, a process known as “inside-out signaling” (49, 50). This inside-out signal involves the activation of SLP76, ADAP, RIAM, and Rap1/RapL. Furthermore, the high affinity configuration of LFA-1 is acquired through an LFA-1-mediated downstream signal (51, 52), known as “outside-in signaling.” This outside-in signal induces activation of kinases and clustering of SLP76/ADAP (53, 54).

**Figure 3:**
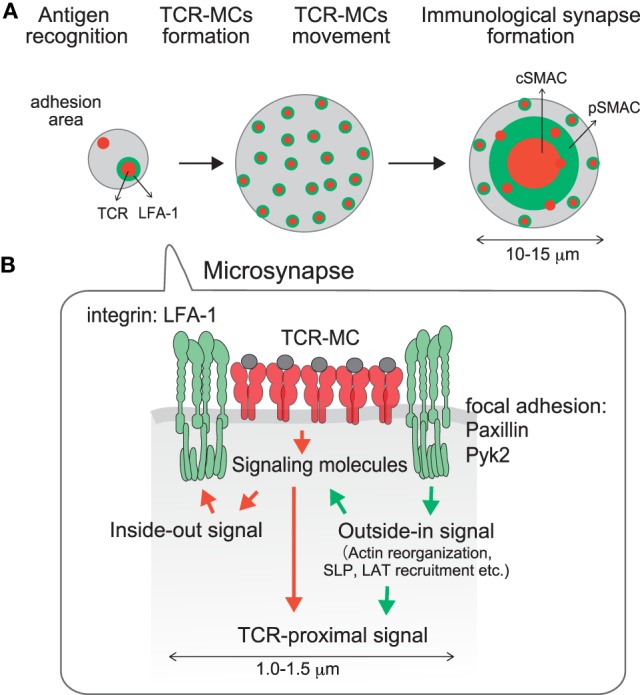
**A microsynapse composed of a core TCR–MC and a surrounding micro adhesion-ring**. **(A)** time course of microsynapse generation, **(B)** molecular assembly in the microsynapse. Immediately after TCR–MCs are formed, an adhesion-ring composed of integrin LFA-1 and focal adhesion molecules such as Paxillin and Pyk2 is generated around the TCR–MC. Because the structure resembles the mature Immunological Synapse in a micro scale, this structure was designated the microsynapse. The adhesion-ring is dependent on LFA-1 outside-in signaling and is supported by F-actin and Myosin II. Cluster formation by LAT and SLP76, but not the TCR or ZAP70, is supported by the microsynapse. The adhesion-ring is a transient structure and disappears before cSMAC formation. The microsynapse is sustained upon weak TCR stimulation, whereas it disappears quickly upon strong stimulation, suggesting that it functions to augment the TCR activation signal upon weak stimulation.

In the mature IS, the cSMAC as the TCR-enriched central region is surrounded by LFA-1 at the peripheral region as the pSMAC, which forms a “bulls-eye” shaped structure. During the course of IS formation, the cSMAC is generated by the translocation of peripherally induced TCR–MCs into the center of the interface. Then how is LFA-1 accumulated into the pSMAC? We found that LFA-1, as well as focal adhesion molecules represented by Pyk2, Paxillin, and vinculin, accumulate just around the TCR–MC and form a kind of “adhesion-ring” in micro scale during the very initial stage of T cell activation (55). The formation of the micro adhesion ring is dependent on LFA1, because no adhesion ring is formed in the absence of the LFA1-ICAM1 interaction on a planar bilayer lacking ICAM-1. The micro adhesion-ring is induced transiently after the initial formation of TCR–MCs, and disappears before the TCR–MCs move to the center to form a cSMAC (Figure 3) (55). The bulls-eye shaped structure with the central TCR–MCs surrounded by the micro adhesion-ring of LFA1, Pyk2, and Paxillin resembles the structure of the mature IS, represented by the central TCR surrounded by LFA1, therefore we suggest naming this structure the “microsynapse” (Figure 3). In addition to LFA1 signals, microsynapse formation is totally dependent on F-actin, since inhibitors of both F-actin and Arp2/3 block the formation of the adhesion ring, but this treatment had no effect on TCR–MC formation. The involvement of Myosin II as an F actin-related effector molecule in microsynapse formation was also analyzed. Treatment with a Myosin II inhibitor reduced microsynapse formation while retaining TCR–MC formation. These observations all indicate that F-actin supports the formation and function of the microsynapse. The functional importance of the microsynapse was revealed by the observation that it is maintained for a longer period when T cells are stimulated with low doses of Ag or weak stimulation, whereas it exists only transiently upon strong stimulation. This was similarly observed upon T cell stimulation with low affinity Ag peptide, such as altered peptide ligand (APL) or with T cells whose TCR had low affinity for the Ag–MHC. These data suggest that the microsynapse structure functions to enhance cellular adhesion to support TCR–MCs, which generate initial activation signals particularly upon weak stimulation. Weak interaction between the TCR and pMHC may require stronger cell adhesion mediated by the microsynapse to induce Ag recognition, followed by triggering initial activation signals. On the other hand, strong TCR engagement can induce sufficient signals for activation by itself in the relative absence of such strong cellular adhesion or co-stimulation.

The requirement for F-actin in microsynapse formation is consistent with the formation of F-actin clusters in TCR–MCs, which are localized at the center of the microsynapse. The F-actin at the TCR–MC is clearly distinct from the peripheral large actin ring and is specialized to support the microsynapse. Whereas the clustering of TCR, CD3, and ZAP70 is relatively independent of F-actin, clustering of LAT and SLP76, as well as molecules in the adhesion-ring such as LFA1, Pyk2 and paxillin, is strongly dependent on F-actin. Therefore, the components of the microsynapse induce two different types of clusters; F-actin dependent clusters (LAT, SLP76, adhesion-ring) and relatively F-actin independent clusters (TCR, ZAP70, etc.). Dependency of F-actin was found to parallel the dependency on TCR signal strength. Whereas TCR and ZAP70 cluster formation is dependent on stimulation signal strength, LAT and SLP76 clusters are relatively independent of signal strength. Such differences in the molecular dynamics of LAT and SLP76 from TCR and ZAP70 are evidence that LAT and SLP76 clustering are dependent on the microsynapse, which is supported by F-actin. Recently it was reported that the phospho-PLCγ cluster is formed in an F-actin- and WASP-dependent manner (41). Since the “actin foci” supporting phospho-PLCγ are quite similar to the F-actin cluster at the center of the microsynapse, the PLCγ cluster may also be supported by the microsynapse.

## Concluding Remarks

Initial contact of a T cell with a cognate Ag-bearing APC induces T cell activation. This critical interaction creates the IS to deliver signals into T cells. The activation unit leading to T cell activation is the TCR–MC, which recruits downstream signaling molecules and mediates the activation signal. The TCR–MC is supported by a ring of adhesion molecules as the microsynapse. Since the cSMAC is formed mainly upon strong stimulation and under limited circumstances *in vivo*, microsynapses generated even upon weak stimulation may play more general and critical roles for early T cell activation in physiological situations. Although at present, the fine analyses described here can be achieved only by using a planar bilayer system, the technique should be extended to the analysis of cell–cell interactions *in vivo* by using *in vivo* imaging microscopy with better resolution. To get a clear picture of the signal events at the IS, first, the cooperative signaling between the TCR–MC signal and other signals such as co-stimulation, adhesion, cytokine, and innate signals should be clarified, and second, individual signaling pathways downstream of the TCR–MC, e.g., Ras-MAPK and PI3K-mTOR, should be analyzed in a spatial-temporal manner, i.e., both the timing and cellular compartmentalization of positive vs. negative signaling molecules need to be studied. The dynamics of these opposing signals may fine tune the activation signals, which controls the direction of cell fate.

## Author Contributions

AH-T performed experiments, and TS and AH-T wrote the review article.

## Conflict of Interest Statement

The authors declare that the research was conducted in the absence of any commercial or financial relationships that could be construed as a potential conflict of interest.
